# Poly[[[μ-1,1′-(butane-1,4-di­yl)diimidazole-κ^2^ *N* ^3^:*N* ^3′^](μ-cyclo­hexane-1,4-dicarboxyl­ato-κ^4^ *O* ^1^,*O* ^1′^:*O* ^4^,*O* ^4′^)nickel(II)] 0.25-hydrate]

**DOI:** 10.1107/S1600536809004024

**Published:** 2009-02-11

**Authors:** Chun-Hui Yang, Guang Yang, Zhen-Wu Du, Jun-Feng Lv, Wei-Tian Yin

**Affiliations:** a The Third Hospital of Jilin University, Changchun 130000, People’s Republic of China; b School of Pharmaceurtical Sciences, Jilin University, Changchun 130000, People’s Republic of China; c The Second Hospital of Jilin University, Changchun 130000, People’s Republic of China

## Abstract

In the title coordination polymer, {[Ni(C_8_H_10_O_4_)(C_10_H_14_N_4_)]·0.25H_2_O}_*n*_, the coordination of the Ni^II^ ion is distorted octa­hedral. The 1,1′-(butane-1,4-di­yl)diimidazole ligand and the cyclo­hexane-1,4-dicarboxyl­ate dianion bridge metal centres, forming a two-dimensional (4,4) network. The network is consolidated by O—H⋯O hydrogen bonds between the statistically occupied water molecules and O atoms of the two carboxylate groups.

## Related literature

For potential applications of metal-organic coordination polymers, see: Yang *et al.* (2008 ▶). For metal-organic networks with diimidazole-containing ligands, see: Batten & Robson (1998 ▶). For flexible ligands such as 1,1′-(butane-1,4-di­yl)diimidazole, see: Ma *et al.* (2003 ▶).
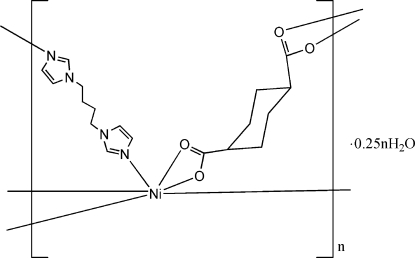


## Experimental

### 

#### Crystal data

[Ni(C_8_H_10_O_4_)(C_10_H_14_N_4_)]·0.25H_2_O
*M* *_r_* = 423.63
Monoclinic, 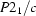

*a* = 9.0045 (9) Å
*b* = 11.9991 (12) Å
*c* = 17.5811 (17) Å
β = 95.755 (2)°
*V* = 1890.0 (3) Å^3^
*Z* = 4
Mo *K*α radiation
μ = 1.06 mm^−1^
*T* = 293 (2) K
0.31 × 0.27 × 0.22 mm

#### Data collection

Bruker APEX CCD area-detector diffractometer
Absorption correction: multi-scan (*SAINT*; Bruker, 1998 ▶) *T*_min_ = 0.711, *T*_max_ = 0.793
10421 measured reflections
3725 independent reflections
2999 reflections with *I* > 2σ(*I*)
*R*_int_ = 0.040

#### Refinement

*R*[*F* ^2^ > 2σ(*F* ^2^)] = 0.040
*wR*(*F* ^2^) = 0.097
*S* = 1.03
3725 reflections
259 parameters
5 restraints
H atoms treated by a mixture of independent and constrained refinement
Δρ_max_ = 0.57 e Å^−3^
Δρ_min_ = −0.27 e Å^−3^

### 

Data collection: *SMART* (Bruker, 1998 ▶); cell refinement: *SAINT* (Bruker, 1998 ▶); data reduction: *SAINT*; program(s) used to solve structure: *SHELXS97* (Sheldrick, 2008 ▶); program(s) used to refine structure: *SHELXL97* (Sheldrick, 2008 ▶); molecular graphics: *SHELXTL* (Sheldrick, 2008 ▶); software used to prepare material for publication: *SHELXTL*.

## Supplementary Material

Crystal structure: contains datablocks global, I. DOI: 10.1107/S1600536809004024/bt2863sup1.cif

Structure factors: contains datablocks I. DOI: 10.1107/S1600536809004024/bt2863Isup2.hkl

Additional supplementary materials:  crystallographic information; 3D view; checkCIF report

## Figures and Tables

**Table 1:** Hydrogen-bond geometry (Å, °)

*D*—H⋯*A*	*D*—H	H⋯*A*	*D*⋯*A*	*D*—H⋯*A*
O1*W*—H*W*12⋯O2^i^	0.87 (2)	1.99 (5)	2.714 (11)	141 (7)
O1*W*—H*W*11⋯O3	0.87 (2)	1.88 (2)	2.663 (12)	149 (4)

Symmetry code: (i) 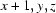.

**Fig. 1.**
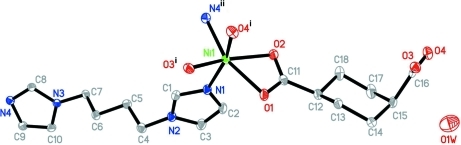
Anisotropic displacement ellipsoid plot at the 30% probability level; hydrogen atoms are drawn as spheres of arbitrary radius; symmetry operations i: 2 - x, 1/2 + y, 1.5 - z; ii: -x, y - 1/2, 1.5 - z).

**Fig. 2.**
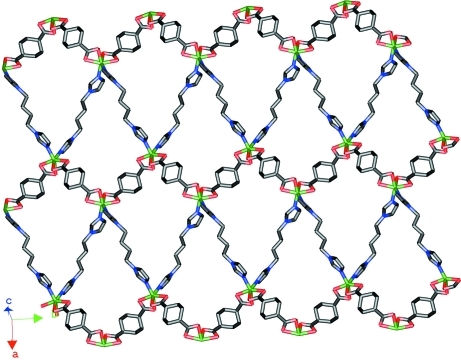
A view of the two-dimensional (4,4) network of (I).

## supplementary crystallographic information

### Comment

Metal-organic coordination polymers are currently of great interest due to their interesting structures and potential applications (Yang *et al.*, 2008). So far, some interesting interpenetrated or entangled metal-organic networks with diimidazole-containing ligands have been documented (Batten & Robson, 1998). Flexible ligands such as 1,1'-(butane-1,4-diyl)diimidazole (*L*) have not been well explored to date (Ma *et al.*, 2003). In this work, we selected cyclohexane-1,4-dicarboxylatic acid (H_2_cdc) and *L* as linkers, generating a new coordination polymer, [Ni(cdc)(*L*)]^.^0.25H_2_O, (I), which is reported here.

In compound (I) each Ni^II^ atom is six-coordinated by two N atoms from two different *L* ligands, and four O atoms from two different cdc ligands in a distorted octohedral coordination sphere. The two neighbouring Ni^II^ atoms are bridged by the cdc and *L* ligands to form a two-dimensional (4,4) network. The O–H···O hydrogen bonds observed in the network consolidated the structure of (I) (Table 1).

### Experimental

A mixture of H_2_cdc (0.5 mmol), *L* (0.5 mmol), NaOH (1 mmol) and NiCl_2_^.^6H_2_O (0.5 mmol) was suspended in 12 ml of deionized water and sealed in a 20-ml Teflon-lined autoclave. Upon heating at 140°C for three days, the autoclave was slowly cooled to room temperature. The crystals were collected, washed with deionized water and dried.

### Refinement

H atoms on C atoms were generated geometrically and refined as riding atoms with C—H= 0.93Å and *U*_iso_(H)= 1.2 times *U*_eq_(C). A peak of 1.9 eÅ^-3^ showed up in the final difference map and was refined as a partially (0.25) occupied water molecule, because the U value went up too high when the O atom was refined as fully occupied. The water H-atoms   were set to forming the best hydrogen bonds. They were refined with distance restraints of O–H = 0.85±0.02 Å; their isotropic displacement parameters were set to  1.5U_eq_(O).

### Crystal data

**Table:**

[Ni(C_8_H_10_O_4_)(C_10_H_14_N_4_)]·0.25H_2_O	*F*(000) = 890
*M**_r_* = 423.63	*D*_x_ = 1.489 Mg m^−^^3^
Monoclinic, *P*2_1_/*c*	Mo *K*α radiation, λ = 0.71073 Å
Hall symbol: -P 2ybc	Cell parameters from 3725 reflections
*a* = 9.0045 (9) Å	θ = 1.1–26.0°
*b* = 11.9991 (12) Å	µ = 1.06 mm^−^^1^
*c* = 17.5811 (17) Å	*T* = 293 K
β = 95.755 (2)°	Block, green
*V* = 1890.0 (3) Å^3^	0.31 × 0.27 × 0.22 mm
*Z* = 4	

### Data collection

**Table:**

Bruker APEX CCD area-detector diffractometer	3725 independent reflections
Radiation source: fine-focus sealed tube	2999 reflections with *I* > 2σ(*I*)
graphite	*R*_int_ = 0.040
φ and ω scans	θ_max_ = 26.0°, θ_min_ = 2.1°
Absorption correction: multi-scan (*SAINT*; Bruker, 1998)	*h* = −11→6
*T*_min_ = 0.711, *T*_max_ = 0.793	*k* = −14→14
10421 measured reflections	*l* = −21→21

### Refinement

**Table:**

Refinement on *F*^2^	Primary atom site location: structure-invariant direct methods
Least-squares matrix: full	Secondary atom site location: difference Fourier map
*R*[*F*^2^ > 2σ(*F*^2^)] = 0.040	Hydrogen site location: inferred from neighbouring sites
*wR*(*F*^2^) = 0.097	H atoms treated by a mixture of independent and constrained refinement
*S* = 1.03	*w* = 1/[σ^2^(*F*_o_^2^) + (0.0493*P*)^2^] where *P* = (*F*_o_^2^ + 2*F*_c_^2^)/3
3725 reflections	(Δ/σ)_max_ = 0.002
259 parameters	Δρ_max_ = 0.57 e Å^−^^3^
5 restraints	Δρ_min_ = −0.27 e Å^−^^3^

### Special details

**Table:**

Geometry. All e.s.d.'s (except the e.s.d. in the dihedral angle between two l.s. planes) are estimated using the full covariance matrix. The cell e.s.d.'s are taken into account individually in the estimation of e.s.d.'s in distances, angles and torsion angles; correlations between e.s.d.'s in cell parameters are only used when they are defined by crystal symmetry. An approximate (isotropic) treatment of cell e.s.d.'s is used for estimating e.s.d.'s involving l.s. planes.
Refinement. Refinement of *F*^2^ against ALL reflections. The weighted *R*-factor *wR* and goodness of fit *S* are based on *F*^2^, conventional *R*-factors *R* are based on *F*, with *F* set to zero for negative *F*^2^. The threshold expression of *F*^2^ > σ(*F*^2^) is used only for calculating *R*-factors(gt) *etc*. and is not relevant to the choice of reflections for refinement. *R*-factors based on *F*^2^ are statistically about twice as large as those based on *F*, and *R*- factors based on ALL data will be even larger.

### Fractional atomic coordinates and isotropic or equivalent isotropic displacement parameters (Å^2^)

**Table:**

	*x*	*y*	*z*	*U*_iso_*/*U*_eq_	Occ. (<1)
C1	0.4322 (3)	0.2316 (2)	0.81343 (15)	0.0268 (6)	
H1	0.4040	0.2702	0.7684	0.032*	
C2	0.5387 (3)	0.1159 (2)	0.89461 (16)	0.0324 (7)	
H2	0.5993	0.0591	0.9162	0.039*	
C3	0.4442 (3)	0.1774 (2)	0.93221 (16)	0.0348 (7)	
H3	0.4277	0.1707	0.9834	0.042*	
C4	0.2636 (3)	0.3356 (2)	0.89436 (17)	0.0299 (6)	
H4A	0.2869	0.4051	0.8700	0.036*	
H4B	0.2666	0.3491	0.9489	0.036*	
C5	0.1088 (3)	0.2991 (2)	0.86426 (15)	0.0266 (6)	
H5A	0.0832	0.2318	0.8906	0.032*	
H5B	0.1070	0.2818	0.8103	0.032*	
C6	−0.0072 (3)	0.3893 (2)	0.87522 (16)	0.0259 (6)	
H6A	0.0055	0.4157	0.9276	0.031*	
H6B	0.0076	0.4518	0.8418	0.031*	
C7	−0.1640 (3)	0.3435 (2)	0.85724 (15)	0.0260 (6)	
H7A	−0.1745	0.3144	0.8055	0.031*	
H7B	−0.1788	0.2823	0.8917	0.031*	
C8	−0.3756 (3)	0.4666 (2)	0.80760 (15)	0.0257 (6)	
H8	−0.3762	0.4445	0.7569	0.031*	
C9	−0.4306 (3)	0.5472 (2)	0.90939 (16)	0.0307 (6)	
H9	−0.4775	0.5923	0.9427	0.037*	
C10	−0.3131 (3)	0.4788 (2)	0.92988 (16)	0.0315 (7)	
H10	−0.2652	0.4686	0.9788	0.038*	
C11	0.8358 (3)	−0.0059 (2)	0.81662 (15)	0.0281 (6)	
C12	0.9464 (3)	−0.0679 (2)	0.87253 (16)	0.0322 (7)	
H12	0.9330	−0.0388	0.9235	0.039*	
C13	1.1072 (3)	−0.0430 (2)	0.85861 (17)	0.0341 (7)	
H13A	1.1218	0.0371	0.8576	0.041*	
H13B	1.1264	−0.0724	0.8092	0.041*	
C14	1.2175 (4)	−0.0939 (2)	0.92035 (17)	0.0426 (8)	
H14A	1.2078	−0.0566	0.9685	0.051*	
H14B	1.3183	−0.0815	0.9072	0.051*	
C15	1.1929 (3)	−0.2187 (2)	0.93014 (16)	0.0365 (7)	
H15	1.2505	−0.2404	0.9781	0.044*	
C16	1.2504 (3)	−0.2892 (2)	0.86740 (15)	0.0270 (6)	
C17	1.0291 (4)	−0.2452 (3)	0.93920 (17)	0.0432 (8)	
H17A	1.0155	−0.3254	0.9388	0.052*	
H17B	1.0048	−0.2175	0.9883	0.052*	
C18	0.9220 (3)	−0.1936 (2)	0.87602 (17)	0.0370 (7)	
H18A	0.8199	−0.2088	0.8858	0.044*	
H18B	0.9385	−0.2269	0.8273	0.044*	
N1	0.5319 (2)	0.14977 (17)	0.81948 (12)	0.0241 (5)	
N2	0.3770 (2)	0.25174 (17)	0.88028 (12)	0.0260 (5)	
N3	−0.2796 (2)	0.42811 (17)	0.86446 (12)	0.0233 (5)	
N4	−0.4696 (2)	0.53970 (17)	0.83217 (12)	0.0243 (5)	
O1	0.8555 (2)	0.09615 (14)	0.80581 (12)	0.0330 (5)	
O2	0.7224 (2)	−0.05498 (15)	0.78388 (10)	0.0297 (4)	
O1W	1.5782 (13)	−0.1622 (11)	0.8909 (6)	0.076 (3)	0.25
HW12	1.597 (7)	−0.104 (6)	0.865 (8)	0.115*	0.25
HW11	1.521 (4)	−0.200 (10)	0.858 (6)	0.115*	0.25
O3	1.3285 (2)	−0.24602 (14)	0.81902 (10)	0.0283 (4)	
O4	1.2237 (2)	−0.39253 (14)	0.86564 (11)	0.0328 (5)	
Ni1	0.65187 (3)	0.09925 (3)	0.733512 (18)	0.02147 (12)	

### Atomic displacement parameters (Å^2^)

**Table:**

	*U*^11^	*U*^22^	*U*^33^	*U*^12^	*U*^13^	*U*^23^
C1	0.0233 (14)	0.0298 (14)	0.0270 (15)	0.0035 (11)	0.0003 (12)	0.0035 (12)
C2	0.0332 (16)	0.0337 (15)	0.0296 (15)	0.0115 (12)	−0.0007 (13)	0.0045 (12)
C3	0.0381 (17)	0.0397 (16)	0.0266 (15)	0.0086 (14)	0.0025 (13)	0.0046 (13)
C4	0.0230 (14)	0.0305 (14)	0.0365 (16)	0.0039 (12)	0.0046 (12)	−0.0050 (12)
C5	0.0243 (14)	0.0287 (14)	0.0272 (15)	0.0011 (11)	0.0048 (12)	−0.0024 (11)
C6	0.0224 (14)	0.0244 (13)	0.0313 (15)	0.0024 (11)	0.0041 (12)	−0.0001 (11)
C7	0.0242 (14)	0.0230 (13)	0.0303 (15)	0.0043 (11)	0.0007 (12)	−0.0027 (11)
C8	0.0243 (14)	0.0284 (14)	0.0241 (14)	0.0022 (11)	0.0009 (12)	−0.0028 (11)
C9	0.0273 (15)	0.0346 (15)	0.0302 (15)	0.0081 (12)	0.0032 (12)	−0.0073 (12)
C10	0.0290 (16)	0.0365 (15)	0.0280 (15)	0.0064 (12)	−0.0017 (12)	−0.0077 (12)
C11	0.0257 (15)	0.0274 (14)	0.0318 (16)	0.0069 (12)	0.0060 (12)	−0.0040 (12)
C12	0.0320 (16)	0.0341 (15)	0.0299 (15)	0.0124 (12)	0.0005 (13)	−0.0042 (12)
C13	0.0291 (16)	0.0247 (14)	0.0464 (18)	0.0060 (12)	−0.0067 (14)	−0.0076 (13)
C14	0.0414 (18)	0.0394 (17)	0.0433 (19)	0.0163 (14)	−0.0137 (15)	−0.0166 (14)
C15	0.0401 (18)	0.0447 (17)	0.0231 (15)	0.0223 (14)	−0.0046 (13)	−0.0018 (13)
C16	0.0215 (14)	0.0299 (14)	0.0277 (15)	0.0083 (11)	−0.0064 (12)	0.0022 (12)
C17	0.050 (2)	0.0512 (19)	0.0307 (17)	0.0219 (16)	0.0155 (15)	0.0141 (14)
C18	0.0321 (17)	0.0372 (16)	0.0432 (18)	0.0090 (13)	0.0115 (14)	0.0138 (14)
N1	0.0179 (11)	0.0246 (11)	0.0298 (13)	0.0026 (9)	0.0018 (10)	0.0000 (9)
N2	0.0202 (11)	0.0275 (11)	0.0305 (13)	0.0030 (9)	0.0034 (10)	−0.0006 (10)
N3	0.0197 (11)	0.0241 (11)	0.0260 (12)	0.0033 (9)	0.0013 (9)	−0.0034 (9)
N4	0.0189 (11)	0.0251 (11)	0.0292 (12)	0.0018 (9)	0.0034 (9)	−0.0022 (9)
O1	0.0229 (10)	0.0256 (10)	0.0488 (13)	0.0049 (8)	−0.0052 (9)	−0.0014 (9)
O2	0.0280 (10)	0.0236 (9)	0.0366 (11)	0.0021 (8)	−0.0016 (9)	−0.0010 (8)
O1W	0.087 (8)	0.089 (9)	0.053 (7)	−0.006 (7)	−0.001 (6)	0.003 (6)
O3	0.0332 (11)	0.0220 (9)	0.0298 (10)	0.0014 (8)	0.0041 (9)	0.0013 (8)
O4	0.0275 (11)	0.0276 (10)	0.0448 (12)	0.0037 (8)	0.0100 (9)	0.0034 (9)
Ni1	0.01688 (19)	0.01940 (18)	0.0281 (2)	−0.00028 (13)	0.00193 (14)	−0.00012 (14)

### Geometric parameters (Å, °)

**Table:**

C1—N1	1.327 (3)	C12—C13	1.522 (4)
C1—N2	1.343 (3)	C12—C18	1.527 (4)
C1—H1	0.9300	C12—H12	0.9800
C2—C3	1.348 (4)	C13—C14	1.524 (4)
C2—N1	1.378 (3)	C13—H13A	0.9700
C2—H2	0.9300	C13—H13B	0.9700
C3—N2	1.373 (3)	C14—C15	1.527 (4)
C3—H3	0.9300	C14—H14A	0.9700
C4—N2	1.472 (3)	C14—H14B	0.9700
C4—C5	1.506 (4)	C15—C16	1.521 (4)
C4—H4A	0.9700	C15—C17	1.533 (4)
C4—H4B	0.9700	C15—H15	0.9800
C5—C6	1.529 (3)	C16—O4	1.262 (3)
C5—H5A	0.9700	C16—O3	1.268 (3)
C5—H5B	0.9700	C16—Ni1^i^	2.455 (3)
C6—C7	1.519 (3)	C17—C18	1.527 (4)
C6—H6A	0.9700	C17—H17A	0.9700
C6—H6B	0.9700	C17—H17B	0.9700
C7—N3	1.469 (3)	C18—H18A	0.9700
C7—H7A	0.9700	C18—H18B	0.9700
C7—H7B	0.9700	N1—Ni1	2.036 (2)
C8—N4	1.322 (3)	N4—Ni1^ii^	2.039 (2)
C8—N3	1.336 (3)	O1—Ni1	2.1237 (18)
C8—H8	0.9300	O2—Ni1	2.1213 (18)
C9—C10	1.359 (4)	O1W—HW12	0.87 (2)
C9—N4	1.371 (3)	O1W—HW11	0.87 (2)
C9—H9	0.9300	O3—Ni1^i^	2.0890 (17)
C10—N3	1.361 (3)	O4—Ni1^i^	2.1668 (19)
C10—H10	0.9300	Ni1—N4^iii^	2.039 (2)
C11—O1	1.254 (3)	Ni1—O3^iv^	2.0890 (17)
C11—O2	1.266 (3)	Ni1—O4^iv^	2.1668 (19)
C11—C12	1.522 (4)	Ni1—C16^iv^	2.455 (3)
C11—Ni1	2.446 (3)		
			
N1—C1—N2	111.7 (2)	C14—C15—C17	111.5 (2)
N1—C1—H1	124.1	C16—C15—H15	106.7
N2—C1—H1	124.1	C14—C15—H15	106.7
C3—C2—N1	109.8 (2)	C17—C15—H15	106.7
C3—C2—H2	125.1	O4—C16—O3	120.2 (2)
N1—C2—H2	125.1	O4—C16—C15	119.1 (3)
C2—C3—N2	106.7 (2)	O3—C16—C15	120.7 (2)
C2—C3—H3	126.7	O4—C16—Ni1^i^	61.85 (14)
N2—C3—H3	126.7	O3—C16—Ni1^i^	58.32 (13)
N2—C4—C5	112.0 (2)	C15—C16—Ni1^i^	178.8 (2)
N2—C4—H4A	109.2	C18—C17—C15	112.6 (2)
C5—C4—H4A	109.2	C18—C17—H17A	109.1
N2—C4—H4B	109.2	C15—C17—H17A	109.1
C5—C4—H4B	109.2	C18—C17—H17B	109.1
H4A—C4—H4B	107.9	C15—C17—H17B	109.1
C4—C5—C6	111.7 (2)	H17A—C17—H17B	107.8
C4—C5—H5A	109.3	C17—C18—C12	110.3 (3)
C6—C5—H5A	109.3	C17—C18—H18A	109.6
C4—C5—H5B	109.3	C12—C18—H18A	109.6
C6—C5—H5B	109.3	C17—C18—H18B	109.6
H5A—C5—H5B	107.9	C12—C18—H18B	109.6
C7—C6—C5	110.5 (2)	H18A—C18—H18B	108.1
C7—C6—H6A	109.5	C1—N1—C2	105.0 (2)
C5—C6—H6A	109.5	C1—N1—Ni1	124.44 (18)
C7—C6—H6B	109.5	C2—N1—Ni1	130.41 (18)
C5—C6—H6B	109.5	C1—N2—C3	106.7 (2)
H6A—C6—H6B	108.1	C1—N2—C4	126.5 (2)
N3—C7—C6	112.6 (2)	C3—N2—C4	126.8 (2)
N3—C7—H7A	109.1	C8—N3—C10	107.2 (2)
C6—C7—H7A	109.1	C8—N3—C7	125.8 (2)
N3—C7—H7B	109.1	C10—N3—C7	126.9 (2)
C6—C7—H7B	109.1	C8—N4—C9	105.0 (2)
H7A—C7—H7B	107.8	C8—N4—Ni1^ii^	123.59 (18)
N4—C8—N3	111.9 (2)	C9—N4—Ni1^ii^	130.52 (17)
N4—C8—H8	124.1	C11—O1—Ni1	88.94 (16)
N3—C8—H8	124.1	C11—O2—Ni1	88.73 (15)
C10—C9—N4	109.8 (2)	HW12—O1W—HW11	102 (3)
C10—C9—H9	125.1	C16—O3—Ni1^i^	90.59 (15)
N4—C9—H9	125.1	C16—O4—Ni1^i^	87.25 (16)
C9—C10—N3	106.2 (2)	N1—Ni1—N4^iii^	93.90 (8)
C9—C10—H10	126.9	N1—Ni1—O3^iv^	97.95 (8)
N3—C10—H10	126.9	N4^iii^—Ni1—O3^iv^	99.21 (8)
O1—C11—O2	120.3 (2)	N1—Ni1—O2	96.24 (8)
O1—C11—C12	118.8 (2)	N4^iii^—Ni1—O2	96.94 (8)
O2—C11—C12	120.8 (2)	O3^iv^—Ni1—O2	157.65 (7)
O1—C11—Ni1	60.23 (14)	N1—Ni1—O1	92.94 (8)
O2—C11—Ni1	60.11 (13)	N4^iii^—Ni1—O1	158.47 (7)
C12—C11—Ni1	176.46 (19)	O3^iv^—Ni1—O1	100.02 (7)
C11—C12—C13	111.8 (2)	O2—Ni1—O1	62.00 (7)
C11—C12—C18	114.9 (2)	N1—Ni1—O4^iv^	159.91 (8)
C13—C12—C18	110.1 (2)	N4^iii^—Ni1—O4^iv^	90.69 (8)
C11—C12—H12	106.5	O3^iv^—Ni1—O4^iv^	61.99 (7)
C13—C12—H12	106.5	O2—Ni1—O4^iv^	102.61 (7)
C18—C12—H12	106.5	O1—Ni1—O4^iv^	89.85 (8)
C12—C13—C14	111.7 (3)	N1—Ni1—C11	95.04 (8)
C12—C13—H13A	109.3	N4^iii^—Ni1—C11	128.02 (9)
C14—C13—H13A	109.3	O3^iv^—Ni1—C11	129.80 (8)
C12—C13—H13B	109.3	O2—Ni1—C11	31.17 (8)
C14—C13—H13B	109.3	O1—Ni1—C11	30.83 (8)
H13A—C13—H13B	107.9	O4^iv^—Ni1—C11	97.49 (8)
C13—C14—C15	112.5 (2)	N1—Ni1—C16^iv^	129.02 (9)
C13—C14—H14A	109.1	N4^iii^—Ni1—C16^iv^	96.21 (8)
C15—C14—H14A	109.1	O3^iv^—Ni1—C16^iv^	31.09 (8)
C13—C14—H14B	109.1	O2—Ni1—C16^iv^	131.55 (8)
C15—C14—H14B	109.1	O1—Ni1—C16^iv^	95.26 (8)
H14A—C14—H14B	107.8	O4^iv^—Ni1—C16^iv^	30.90 (8)
C16—C15—C14	113.6 (3)	C11—Ni1—C16^iv^	116.26 (9)
C16—C15—C17	111.2 (3)		

Symmetry codes: (i) −*x*+2, *y*−1/2, −*z*+3/2; (ii) −*x*, *y*+1/2, −*z*+3/2; (iii) −*x*, *y*−1/2, −*z*+3/2; (iv) −*x*+2, *y*+1/2, −*z*+3/2.

### Hydrogen-bond geometry (Å, °)

**Table:**

*D*—H···*A*	*D*—H	H···*A*	*D*···*A*	*D*—H···*A*
O1W—HW12···O2^v^	0.87 (2)	1.99 (5)	2.714 (11)	141 (7)
O1W—HW11···O3	0.87 (2)	1.88 (2)	2.663 (12)	149 (4)

Symmetry codes: (v) *x*+1, *y*, *z*.
